# Cancer immunotherapy is accompanied by distinct metabolic patterns in primary and secondary lymphoid organs observed by non-invasive *in vivo* ^18^F-FDG-PET

**DOI:** 10.7150/thno.35989

**Published:** 2020-01-01

**Authors:** Johannes Schwenck, Barbara Schörg, Francesco Fiz, Dominik Sonanini, Andrea Forschner, Thomas Eigentler, Benjamin Weide, Manuela Martella, Irene Gonzalez-Menendez, Cristina Campi, Gianmario Sambuceti, Ferdinand Seith, Leticia Quintanilla-Martinez, Claus Garbe, Christina Pfannenberg, Martin Röcken, Christian la Fougere, Bernd J Pichler, Manfred Kneilling

**Affiliations:** 1 Department of Nuclear Medicine and Clinical Molecular Imaging, Eberhard Karls University, 72076 Tübingen, Germany; 2 Werner Siemens Imaging Center, Department of Preclinical Imaging and Radiopharmacy, Eberhard Karls University, 72076 Tübingen, Germany; 3 Cluster of Excellence iFIT (EXC 2180) "Image-Guided and Functionally Instructed Tumor Therapies", Eberhard Karls University, 72076 Tübingen, Germany; 4 Department of Internal Medicine, University of Genoa, Italy; 5 Department of Internal Medicine II, Eberhard Karls University, 72076 Tübingen, Germany; 6 Department of Dermatology, Eberhard Karls University, 72076 Tübingen, Germany; 7 Institute of Pathology and Neuropathology and Comprehensive Cancer Center Tübingen, Eberhard Karls University, 72076 Tübingen, Germany; 8 Department of Mathematics "Tullio Levi-Civita", University of Padua, Italy; 9 Nuclear Medicine Unit, Department of Health Sciences, University of Genoa, Italy; 10 Department of Diagnostic and Interventional Radiology, Eberhard Karls University, 72076 Tübingen, Germany; 11 German Cancer Consortium (DKTK), German Cancer Research Center (DKFZ) Partner Site Tübingen, 72076 Tübingen, Germany

**Keywords:** Checkpoint inhibitor therapy, response assessment of immunotherapy, PET/CT, translational, imaging of primary and secondary lymphatic organs

## Abstract

**Purpose:** Cancer immunotherapy depends on a systemic immune response, but the basic underlying mechanisms are still largely unknown. Despite the very successful and widespread use of checkpoint inhibitors in the clinic, the majority of cancer patients do not benefit from this type of treatment. In this translational study, we investigated whether noninvasive *in vivo* positron emission tomography (PET) imaging using 2-[^18^F]fluoro-2-deoxy-D-glucose (^18^F-FDG) is capable of detecting immunotherapy-associated metabolic changes in the primary and secondary lymphoid organs and whether this detection enables the prediction of a successful anti-cancer immune response.

**Methods:** RIP1-Tag2 mice with progressed endogenous insular cell carcinomas underwent a combined cancer immunotherapy consisting of CD4^+^ T cells plus monoclonal antibodies (mAbs) against programmed death ligand-1 (PD-L1) and lymphocyte activation gene-3 (LAG-3) or a sham treatment after radiation-mediated immune cell depletion. A second cohort of RIP1-Tag2 mice underwent exclusive checkpoint inhibitor therapy (CIT) using anti-PD-L1/LAG-3 mAbs or sham treatment without initial immune cell depletion to mimic the clinical situation.

All mice were monitored by ^18^F-FDG-PET combined with anatomical magnetic resonance imaging (MRI). In addition, we retrospectively analyzed PET / computed tomography (CT) scans (PET/CT) regarding ^18^F-FDG uptake of CIT-treated metastatic melanoma patients in the spleen (n=23) and bone marrow (BM; n=20) as well as blood parameters (n=17-21).

**Results:** RIP1-Tag2 mice with advanced insular cell carcinomas treated with combination immunotherapy exhibited significantly increased ^18^F-FDG uptake in the spleen compared to sham-treated mice. Histopathology of the spleens from treated mice revealed atrophy of the white pulp with fewer germinal centers and an expanded red pulp with hyperplasia of neutrophils than those of sham-treated mice. Immunohistochemistry and flow cytometry analyses of the spleens revealed a lower number of T cells and a higher number of neutrophils compared to those in the spleens of sham-treated mice. Flow cytometry of the BM showed enhanced activation of T cells following the treatment schemes that included checkpoint inhibitors. The ratio of ^18^F-FDG uptake at baseline to the uptake at follow-up in the spleens of exclusively CIT-treated RIP1-Tag2 mice was significantly enhanced, but the ratio was not enhanced in the spleens of the sham-treated littermates. Flow cytometry analysis confirmed a reduced number of T cells in the spleens of exclusively CIT-treated mice compared to that of sham-treated mice. A retrospective analysis of clinical ^18^F-FDG-PET/CT scans revealed enhanced ^18^F-FDG uptake in the spleens of some successfully CIT-treated patients with metastatic melanoma, but there were no significant differences between responders and non-responders. The analysis of the BM in clinical ^18^F-FDG-PET/CT scans with a computational segmentation tool revealed significantly higher baseline ^18^F-FDG uptake in patients who responded to CIT than in non-responders, and this relationship was independent of bone metastasis, even in the baseline scan.

**Conclusions:** Thus, we are presenting the first translational study of solid tumors focusing on the metabolic patterns of primary and secondary lymphoid organs induced by the systemic immune response after CIT. We demonstrate that the widely available ^18^F-FDG-PET modality is an applicable translational tool that has high potential to stratify patients at an early time point.

## Introduction

After many years of being explored in experimental studies, immunotherapeutic approaches against cancer are now successfully applied in routine clinical practice 1. Currently, checkpoint inhibitor therapy (CIT) applying the immune checkpoint-specific antibodies against the cytotoxic T-lymphocyte-associated protein-4 (CTLA-4; ipilimumab) and programmed cell death protein-1 (PD-1; nivolumab and pembrolizumab) are approved as first-line treatments for patients with metastatic melanoma 2. Nevertheless, only a portion of these patients benefit from CIT, and many patients suffer from severe side effects independent of treatment success or failure 3. CIT with CTLA-4-blocking mAbs is efficient in approximately 20 % of patients with metastatic melanoma, and with PD-1 mAbs in approximately 40 % of patients. Combining CTLA-4 mAbs with PD-1 mAbs significantly increases the treatment response rate to 60 %, but the combination leads to more severe side effects. Nevertheless, 40-80 % of metastatic melanoma patients do not respond to CIT, and half of the non-responders progress rapidly 4, 5. Consequently, as the window of opportunity to change a treatment protocol for patients with rapidly progressing metastatic melanoma is very narrow, the early identification of CIT responders is essential. Additionally, early identification of non-responders would help to limit the tremendous costs of CIT treatment that threatens the health care systems 6, 7.

There is growing evidence that successful cancer immunotherapy requires a systemic anti-tumor response involving primary (e.g. the bone marrow) and secondary lymphoid organs (e.g. the spleen) as well as a complex interplay between different immune cell populations 8. Unfortunately, the exact mechanisms and dynamics are still unclear. To date, there is no reliable method available for the *in vivo* assessment of successful anticancer immune responses, which could provide treatment stratification of patients which are responding to checkpoint inhibitor treatment 9. Different molecular analysis methods, such as mutational load and PD-L1 expression, have been proven as valuable predictive biomarkers but apply only to a minority of patients 10. However, these methods require usable tissue material, derived from invasive biopsies or resection of primary tumors, and do not take tumor heterogeneity into account.

Molecular imaging, such as positron emission tomography (PET), enables the temporal and spatial quantification of target-specific molecular probes. PET with the glucose analog ^18^F-FDG is widely used in the clinical routine to detect primary tumors and metastases, e.g., melanomas 11.

As immune cells, such as T cells, undergo specific metabolic changes upon activation and quickly switch to extensive glycolysis 12, we employed ^18^F-FDG-PET to identify the metabolic patterns induced by a successful immune response against tumors. In RIP1-Tag2 mice, a well-established tumor model of endogenous insular cell carcinomas 13, *in vivo* ^18^F-FDG PET/MRI identified metabolic activation of the spleen caused by the immunotherapy using anti-PD-L1/anti-LAG-3 mAbs combined with (combo) or without (CIT) the adoptive transfer of tumor antigen-specific Th1 cells. To evaluate whether our preclinical findings could potentially be translated into the clinic, we retrospectively investigated the ^18^F-FDG PET/CT scans of metastatic melanoma patients before and after the onset of checkpoint inhibitor therapy.

## Materials and Methods

### Animals

**Figure 1:**
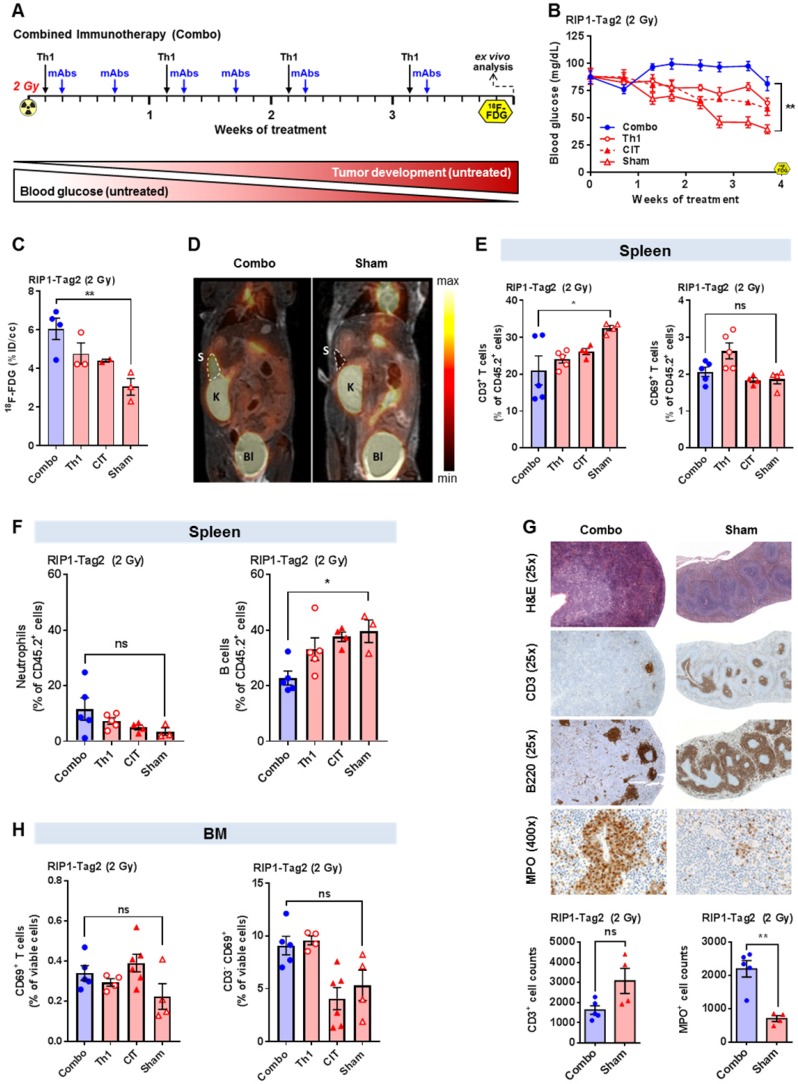
** Investigation of the splenic ^18^F-FDG uptake and splenic immune cell Infiltrate in RIP1-Tag2 mice 4 weeks after the onset of combination immunotherapy. (A)** Combination immunotherapy (combo) treatment scheme for RIP1-Tag2 mice with advanced endogenous insular cell carcinomas. **(B)** Tumor progression was monitored via BGL in RIP1-Tag2 mice. **(C)** Splenic ^18^F-FDG uptake was determined by PET/MRI in combo- (n=4) and sham-treated (n=3) RIP1-Tag2 mice following 4 weeks of treatment and compared to the sham-treated mice (n=3). **(D)** Representative PET/MRI images of ^18^F-FDG uptake in the spleens of mice after 4 weeks of treatment. PET images were fused to MR images for anatomical coregistration. S = spleen, K = kidney, Bl = Bladder. **(E, F)** Flow cytometry analysis of splenic T cells (CD3), B cells (B220), neutrophils and the lymphocyte activation marker CD69 four weeks after the onset of treatment. **(G)** H&E and immunohistochemical staining of spleen slices revealed atrophy of the white pulp with fewer germinal centers in combo-treated mice than in sham-treated mice and a clear expansion of the red pulp with a strongly enhanced accumulation of neutrophils. Quantification of CD3^+^ T cells and MPO^+^ neutrophils in the spleens of combo-treated mice (n=5) compared to those of the sham-treated littermates (n=4). **(H)** Flow cytometry analysis of the BM focusing on CD69-expressing lymphocytes (left) in the combo- and CIT-treated experimental groups. The expression of CD69 by CD3-negative cells exclusively in combo- or single Th1-treated RIP1-Tag2 mice (right). Data are expressed as the mean ± SEM. Each data point represents one mouse (B-H) or the sum of three fields of one tissue slice (200x magnification). (*P<0.05, **P<0.01, ns = not significant).

**Figure 2:**
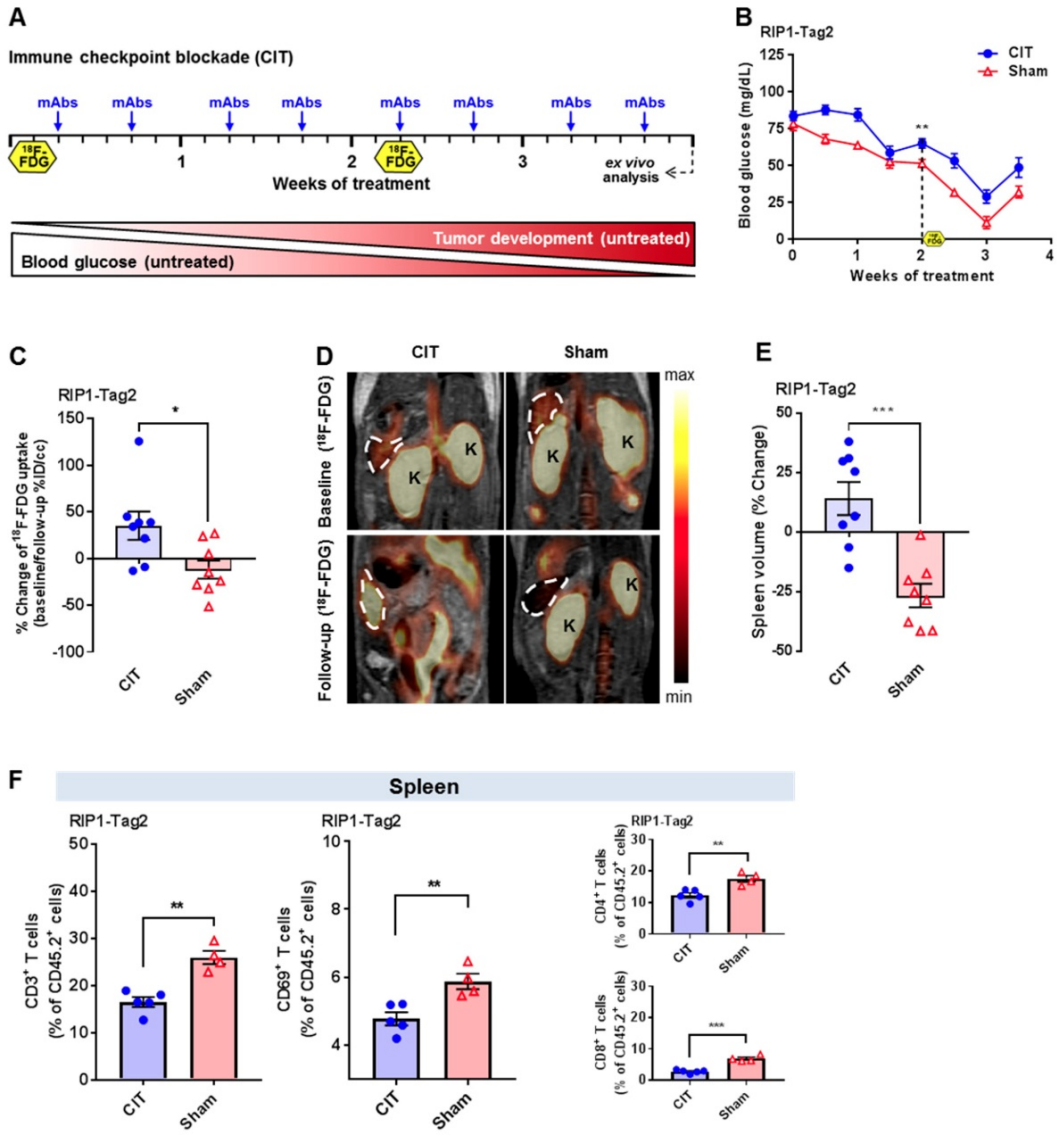
** Investigation of the splenic ^18^F-FDG uptake and splenic immune cell infiltrate of RIP1-Tag2 mice with CIT. (A)** Treatment scheme including two PET investigations before and during the treatment blockade of non-irradiated RIP1-Tag2 mice bearing advanced endogenous insular cell carcinomas. **(B)** Treatment monitoring via the median blood glucose levels in RIP1-Tag2 mice (n=8). **(C)** Changes in the uptake of ^18^F-FDG in the spleens of mice measured by *in vivo* PET between the baseline and follow-up PET scans. **(D)** Representative PET/MRI images showing the ^18^F-FDG uptake in the spleens of RIP1-Tag2 mice at the baseline (upper panel) and at the follow-up (lower panel) PET scans. Dashed line = spleen, K = kidney. **(E)** Changes in the spleen volumes of RIP1-Tag2 mice between the baseline and follow-up scans. CIT increased the spleen volume of RIP1-Tag2 mice after 4 injections of the antibody cocktail. **(F)** Flow cytometry analysis of the spleens after 4 weeks of CIT revealed less splenic CD4^+^ and CD8^+^ T cells as well as less expression of the activation marker CD69 compared to those at baseline (CD4+, CIT: 12.3±0.9 % of CD45.2+ cells; sham: 17.6±1.0 % of CD45.2+ cells, p<0.01; CD8+, CIT: 2.9±0.3 % of CD45.2+ cells; sham: 7.0±0.5 % of CD45.2+ cells, p<0.001). Data are expressed as the mean ± SEM. Each data point represents one mouse (*P<0.05, **P<0.01, ***P<0.001).

Transgenic RIP1-Tag2 mice and transgenic Tag2-TCR mice, both on a C3H background 13, were bred and maintained under specific pathogen-free conditions. All animal experiments were performed according to the German Animal Protection Law with permission from the local authorities (Regierungspräsidium Tübingen, Germany). The therapeutic approaches are outlined in Figure 1A and 2A as well as described in detail in the supplementary material.

### Histopathology and immunohistochemistry of the spleen

The spleens of the first cohort of combo-, CIT-, Th1- or sham-treated RIP1-Tag2 mice were isolated after four weeks of treatment and fixed in 4 % formalin. Serial sections (3-5 µm thick) of the paraffin-embedded tissue were stained with hematoxylin and eosin (H&E). Immunohistochemistry staining for CD3 (SP7, DCS, 1:200, Hamburg, Germany), B220 (BD Biosciences, 1:50, NJ, USA), and myeloperoxidase (MPO, Thermo Scientific, ready-to-use, Fremont, USA) was performed on an automated immunostainer (Discovery, Ventana Medical Systems, Roche Diagnostics, Germany) according to the company's protocols for open procedures with slight modifications. For quantification, CD3- and MPO-positive cells were manually counted in three different fields with a 20x objective (200x magnification) (combo-treated: n=5; sham-treated: n=4).

### Preclinical PET and MR imaging and data analysis

The PET and MR images of the first cohort of the combo- (n=4), CIT- (n=2), Th1- (n=3) or sham-treated (n=3) RIP1-Tag2 mice were acquired four days after the final mAb administration (Figure 1A) using a dedicated small-animal PET scanner (Siemens Preclinical Solutions; Knoxville, USA) and a 7 T small-animal MRI (Bruker Biospin MRI; Germany). The second cohort of CIT- (n=8) or sham-treated (n=8) RIP1-Tag2 mice underwent a baseline PET scan one day before the onset of treatment and a second PET scan four days after the fourth mAb administration (Figure 2A).

After a fasting period, the RIP1-Tag2 mice were intravenously (*i.v.*) injected with 12.5 MBq ^18^F-FDG. Static PET scans were acquired 45 min after the tracer injection for a single time frame of 15 min. The tracer injections, tracer uptake and PET and MRI scans were carried out under 1.5 % isoflurane anesthesia (mixed with 100 % oxygen) using a dedicated vaporizer (Vetland, Louisville, USA) and heat lamps.

Volumes of interest (VOIs) of the spleens were created based on the MR images before they were fused to the respective PET scans using Inveon Reserch Workplace (Siemens Preclinical Solutions). The investigator who conducted the analysis was not aware of the assignment of mice to the experimental groups.

The percent injected dose (%ID) was calculated based on the ^18^F-FDG Uptake (Bq/mL) in the VOIs after correction for the radioactive decay. For a visual comparison between the PET images in Figure 1D and 2D, the signal intensity as well as the color scale of the images have been adjusted with respect to each other.

### Flow cytometry analysis

The spleens of the first cohort of the combo- (n=5), CIT- (n=4), Th1- (n=5) or sham-treated (n=4) RIP1-Tag2 mice as well as the spleens of the second cohort of the CIT- (n=5) or sham-treated (n=4) of RIP1Tag2 mice were isolated after four weeks of treatment for flow cytometry analysis. The BM from RIP1-Tag2 mice was isolated at a similar time point deriving from another trial (combo-treated: n=5, Th1-treated: n=4, CIT-treated: n=6; sham-treated: n=4). Freshly prepared single cell suspensions from the spleens or BM were labeled *ex vivo* using the following fluorescent monoclonal antibodies: (V500)-CD45.2 (BD Bioscience, NJ, USA), (V450)-CD3 (spleen, BD Bioscience), (FITC)-CD3 (BM; Biolegend, CA, USA), (FITC)-CD8 (BD Bioscience), (APC-Cy7)-CD4 (Thermo Fisher, MA, USA), (PE-Cy7)-CD69 (spleen; Thermo Fisher), (BV650)-CD69 (BM; Biolegend), (FITC)-B220 (Thermo Fisher), (PE)-Ly6G (BD Biosciences) and (PE-Cy7)-CD11b (BD Biosciences); the samples were analyzed by multicolor flow cytometry (LSR Fortessa; BD Biosciences). Fluorescence data were analyzed via FlowJo (FlowJo, LLC, OR, USA).

### Patient cohort

In total, the data from 39 patients with metastatic melanoma and their ^18^F-FDG-PET/CT scans before and after the start of therapy with CTLA-4 or PD-1 mAbs were available from a register study at the University Hospital of Tübingen (064 / 2013 BO1). For the analysis of the spleen, patients who had a second malignant disease, surgical resections of metastasis, or splenic metastasis were excluded. Additionally, we excluded uveal melanomas due to their different biological behavior (Table S1). In total, 23 patients with ^18^F-FDG-PET scans within 50 days before and 125 days after the start of therapy were identified for further analysis (Table S2: 14 responder: 3x nivolumab, 6x pembrolizumab, 4x ipilimumab, 1x ipilimumab + nivolumab; 9 non-responder: 1x nivolumab, 4x pembrolizumab, 5x ipilimumab).

For the analysis of the bone marrow, we excluded patients with diffuse bone metastases, bone marrow carcinosis, and previous external beam radiation therapy on bone marrow regions (Table S3: n=3; 1x responder; 2x non-responder).

The used segmentation program is able to recognize and exclude bone metastases within the trabecular volume automatically. However, to test the possible influence of tracer spillover from tumor localizations into the trabecular bone, we conducted all computations of trabecular bone activity before and after removing patients with bone metastasis. An overview about the patient selection is displayed in Figure S1.

### Analysis of the clinical splenic ^18^F-FDG-PET/CT data

The PET scans were acquired on a Siemens Biograph PET/CT Scanner (Siemens Healthcare, Knoxville, USA) 60 min after the *i.v.* application of approximately 300 MBq ^18^F-FDG using a clinical protocol according to the in-house standards, which included a fully diagnostic contrast-enhanced CT scan (portal venous phase; 120 mL, 2.0 mL/s; Ultravist; Bayer, Leverkusen, Germany). The examined field ranged from the orbitomeatal line to the mid-thigh. The scans were performed with elevated arms. The PET data were reconstructed using an ultra-HD reconstruction including time-of-flight and point-spread functions (2 iterations, 21 subsets, Gaussian filter 2 mm) and were corrected for attenuation as well as scatter. Experienced physicians with long-standing expertise in hybrid imaging visually segmented visually the whole spleen in the CT images and copied the VOI to the coregistered PET data for semiquantitative analysis. The SUV_mean_ was calculated using the Hermes Hybrid Viewer software (Hermes Medical Solutions, Stockholm, Sweden).

### Computational analysis of the bone marrow activity

To identify and estimate the metabolic activation of the bone marrow, we applied a computational analysis to the hybrid ^18^F-FDG-PET/CT images. The software application is based on a segmentation analysis method and isolates the signal of the trabecular bone from that of the surrounding tissues, in particular, from the cortical bone 14-16. Briefly, the algorithm identifies the bone border on CT images by recognizing the sharp attenuation contrast between soft tissues and the outer cortical bone as well as the boundary between the inner cortical bone and trabecular bone. After defining the trabecular bone volume, the program generates a mask, which is then transported onto the PET dataset to extract the functional information from the voxels belonging to the trabecular bone. To minimize the possible confounding factors, the mean SUV from the trabecular bone was normalized to the blood pool activity (10-slice VOI on the inferior vena cava) to obtain the target-to-background ratio (TBR) 17.

Separate analyses were conducted for the whole-body skeleton (entire skeletal system in the field of view of the acquired PET scans), the axial skeleton (all vertebrae and the sternum), and the appendicular bones (humerus- and upper half of the femur shafts).

### Blood parameters from patients

Patients were selected as previously described. The investigated blood parameters were obtained from the laboratory values measured in the context of the clinical routine at the University Hospital Tübingen.

### Statistical analysis

Blood glucose levels (BGL) were compared within the cohorts at the time point shortly before the follow-up ^18^F-FDG PET scans. The preclinical *in vivo* and *ex vivo* data and the clinical data derived from patients (^18^F-FDG uptake, blood parameters and TBR values between responders and non-responders) were compared using unpaired, two-tailed Student's t-tests. Data are displayed as the mean ± SEM. Significance is indicated in the figures by *p<0.05, **p<0.01 and ***p<0.001. The statistical analyses were performed using GraphPad Prism (GraphPad Software, CA, USA).

## Results

### Preclinical combination cancer immunotherapy in mice with advanced cancer

At the onset of treatment, all RIP1-Tag2 mice had already developed progressed insula cell carcinomas in the pancreas as indicated by low blood glucose levels (BGL; inversely correlates with tumor progression 13) of approximately 80 mg/dl, whereas healthy wild-type mice usually display BGLs of approximately 120 mg/dl. Our new established, highly effective combo-treatment (Figure 1A) was able to significantly increase and stabilize the BGL after 4 weeks of treatment when compared to the sham-treatment (p<0.01; Figure 1B). The Tag2-Th1 cell treatment without CIT or CIT-treatment without Tag2-Th1 cells was less efficient than the combo-treatment but nevertheless was superior to the sham-treatment at the end of the experiment (Figure 1B).

### Combination cancer immunotherapy enhances the glucose metabolism of the spleen

Next, we focused on the impact of our different treatments on the glucose metabolism in the primary and secondary lymphoid organs of RIP1-Tag2 mice, as measured by ^18^F-FDG PET. After 4 weeks, we observed significantly higher ^18^F-FDG uptake in the spleens of combo-treated RIP1-Tag2 mice than in the spleens of sham-treated mice (p<0.01) (Figure 1C/D). However, as a consequence of the limited spatial resolution, we were unable to analyze the ^18^F-FDG uptake in the lymph nodes or bone marrow of the experimental mice.

Furthermore, we aimed to identify the cellular origin of the enhanced glucose metabolism in the spleens of RIP1-Tag2 mice. We expected that the enhanced PET signal was the consequence of an enlarged, highly activated and proliferating T cells population in the spleen. Interestingly, flow cytometry analysis revealed a significantly decreased population of CD3+ T cells in the spleens of combo-treated mice compared to the population in the spleens of sham-treated RIP1-Tag2 mice (p<0.05; Figure 1E).

When focusing on activated CD3^+^ T cells, flow cytometry analysis revealed a slightly enhanced CD69 expression in the exclusively Th1-treated mice but not in the combo-, CIT- or sham-treated mice (Figure 1E). Furthermore, we determined a trend towards a higher number of neutrophils and a significantly lower number of B cells in the spleens of combo-treated mice than in the spleens of sham-treated mice (p<0.05; Figure 1F).

To gain deeper insights into the combo-treatment-induced changes in spleen morphology and the distribution of T cells, B cells and neutrophils, we conducted H&E and immunohistochemical staining (for CD3, B220, MPO). We observed atrophy of the white pulp with fewer germinal centers in the spleens of combo-treated mice than in the spleens of sham-treated mice (CD3 and B220-stained sections, Figure 1G). Additionally, we determined a clear expansion of the red pulp with a strongly enhanced accumulation of neutrophils mainly surrounding the sinusoids of the red pulp (Figure 1G). In agreement with the data obtained from flow cytometry, the CD3 immunohistochemistry revealed an unexpected reduction in the number of CD3^+^ T cells within the spleens of combo-treated RIP1-Tag2 mice than in the spleens of sham-treated mice. MPO immunohistochemistry highlighted a strong increase in the number of MPO-expressing immune cells in the spleens of combo-treated mice compared to that in the spleens of sham-treated mice, indicating that the combo- treatment might induce an intense systemic humoral immune response associated with a change in the splenic morphology. The quantification of CD3^+^ T cells and MPO^+^ neutrophils revealed a tendency towards the sham-treated mice having half the number of CD3^+^ T cells as the combo-treated (Figure 1G, lower graph); additionally, there was a statistically significant 4-fold increase in the number of MPO^+^ neutrophils in the spleens of the combo-treated littermates when compared to sham-treated mice (p<0.01; Figure 1G, lower graph).

### Combination immunotherapy causes bone marrow activation

To uncover whether combo-, single Th1- or single CIT-treatment affects the primary lymphatic organs, we conducted flow cytometry analysis of the BM of all experimental groups and determined a slightly enhanced number of CD69-expressing T cells following CIT treatment with or without Tag2-Th1 cells (CIT) when compared to sham treatment (Figure 1H, left graph). When focusing exclusively on CD3-negative cells, we determined a slightly enhanced expression of CD69 exclusively in the mice treated with Tag2-Th1 cells in combination with or without immune checkpoint blockade (Th1) compared to sham-treated mice, indicating that CIT might predominantly induce the activation of T cells and that the Tag2-Th1 cells might predominantly induce the activation of B cells (Figure 1H, right graph).

### Preclinical CIT-treatment in mice with advanced cancer similar to the setting in the clinic

We aimed to mimic the clinical conditions without initial radiation and adoptive transfer of tumor antigen-specific T cells. Thus, in the second experimental setup, RIP1-Tag2 mice exclusively underwent CIT treatment without an initial immune cell depletion or Tag2-Th1 cell transfer (Figure 2A). In addition, we conducted ^18^F-FDG-PET/MRI investigations one day before the onset of treatment (baseline) and four days after the 4^th^ administration of the mAbs (follow-up; Figure 2A). CIT treatment was able to induce a moderate but significant therapeutic effect on the BGLs of RIP1-Tag2 mice when compared to sham-treated mice during the course of treatment until the second ^18^F-FDG examination (p<0.01; Figure 2B).

### CIT enhances the glucose metabolism of the spleen

*In vivo* ^18^F-FDG-PET/MRI scans revealed a significant increase in splenic ^18^F-FDG uptake from baseline to follow-up in CIT-treated mice when compared to that of sham-treated mice (p<0.05; Figure 2C/D). Moreover, we determined a significant increase in spleen volume between the baseline and follow-up scans in the CIT-treated RIP1-Tag2 mice and a decrease in spleen volume in sham-treated mice (p<0.001; Figure 2E). Finally, we conducted flow cytometry analysis of the spleens of both experimental groups and determined that the results were in line with our previous results (Figure 1E). Thus, we determined a significantly reduced number of CD3^+^ T cells in the spleens of the CIT-treated mice when compared to the spleens of the sham-treated littermates (p<0.01; Figure 2F). Interestingly, as indicated by CD69 expression, the CD3^+^ T cells were less activated after CIT-treatment than after sham-treatment (p<0.01; Figure 2F). Differential analysis of CD4^+^ and CD8^+^ T cell populations revealed that both populations in the spleens of the CIT-treated group were significantly reduced, in a similar manner, compared to those in the spleens of the sham-treated group (Figure 2F).

### Retrospective analysis of clinical ^18^F-FDG-PET/CT scans with a focus on the spleen

**Figure 3:**
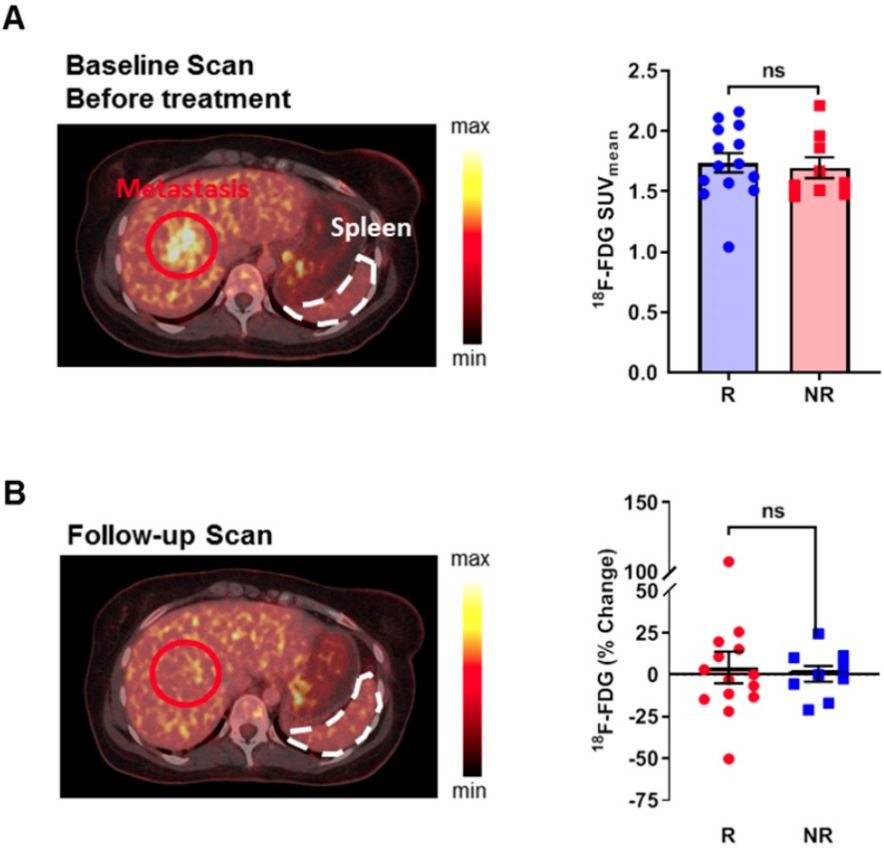
** Retrospective analysis of ^18^F-FDG uptake in the spleen of patients with metastasized melanoma during checkpoint inhibitor treatment. (A)** ^18^F-FDG uptake between responders and non-responders at baseline before the start of treatment. A representative sample image from a patient who responded to treatment displays the physiological ^18^F-FDG uptake in the spleen. **(B)** ^18^F-FDG uptake at the follow-up PET scan. The sample image represents a patient who responded to checkpoint inhibitor therapy and had a slight increase in ^18^F-FDG uptake and an increase in spleen volume after therapy compared to before therapy (compared to 3A; identical patient). Data are expressed as the mean ± SEM (responder n=14; non-responder n=9). Each data point represents one patient (ns = not significant).

To test whether our preclinical results are also valid in CIT-treated patients, we retrospectively analyzed the ^18^F-FDG-PET/CT data from patients with metastatic melanoma. We identified 23 patients who underwent ^18^F-FDG-PET/CT imaging within 50 days before and 125 days after the onset of CTLA-4 or PD-1 mAb treatment. The patients were divided into treatment responders or non-responders according to the clinical decisions made by the treating physicians, which led to the continuation or discontinuation of the CIT-treatment. No significant differences in age were identified between the two groups. One of the non-responders underwent cytotoxic chemotherapy 11 months before the onset of CIT because of an ovarian carcinoma; none of the other patients underwent chemotherapy regimens due to metastatic melanoma. Four of the non-responders (44 %) and 2 of the responders (14 %) previously received external beam irradiation of the non-bone marrow areas. Two of the responders (14 %) and 4 of the non-responders (44%) underwent interferon-alpha therapy; one of the responders and four of the non-responders were treated with a kinase inhibitor before CIT. The baseline ^18^F-FDG-PET/CT scans before CIT revealed no differences in the splenic ^18^F-FDG uptake between responders and non-responders (Figure 3A; Table S2). In line with our preclinical studies, the follow-up ^18^F-FDG-PET/CT scans revealed a tendency towards some patients having high ^18^F-FDG uptake in the spleen, but no significant differences were found between responders and non-responders when comparing the baseline ^18^F-FDG-PET/CT scans to the follow-up scans (Figure 3B).

### Retrospective computational analysis of the ^18^F-FDG uptake in the BM

**Figure 4:**
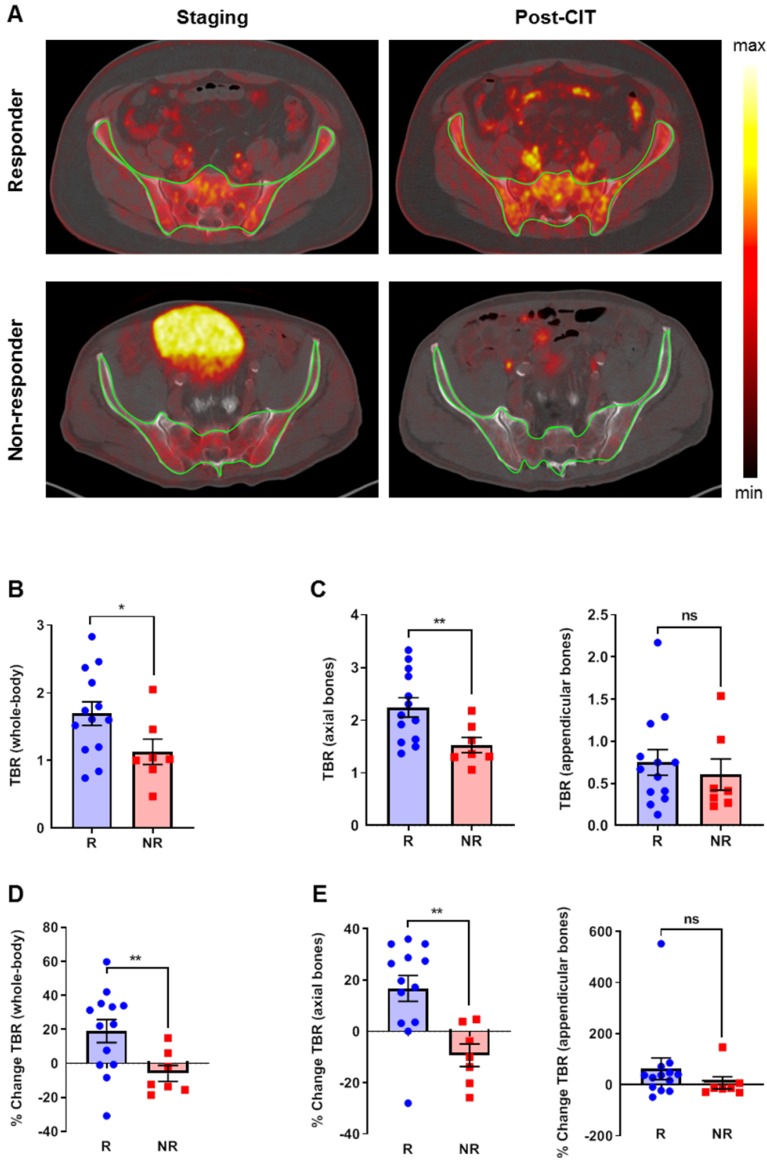
** Retrospective analysis of clinical PET/CT data from the bone marrow using an automatic segmentation tool. (A)** Upper PET images: representative baseline ^18^F-FDG-PET scan (left) and the follow-up ^18^F-FDG-PET scan (right) after the initiation of CIT; the clearly enhanced ^18^F-FDG uptake within the bone is a consequence of successful treatment. Lower PET images: representative baseline ^18^F-FDG-PET scan (left) and the follow-up ^18^F-FDG-PET scan (right) after the initiation of CIT; the rather reduced ^18^F-FDG uptake within the bone is a consequence of CIT failure. **(B)** TBRs at baseline. **(C)** Separate analysis of the axial and appendicular bones. **(D)** Percentage change of the ^18^F-FDG uptake in the whole-body TBR using the ^18^F-FDG-PET/CT scans. **(E)** Percentage change of the ^18^F-FDG uptake after a separate analysis of the axial and appendicular bones. Data are expressed as the mean ± SEM (responder n=13; non-responder n=7). Each data point represents one patient (*P<0.05, **P<0.01, ***P<0.01, ns = not significant).

To assess the impact of CIT on BM, we used an established tool to segment the trabecular bone from the cortical bone for analysis on ^18^F-FDG-PET/CT-scans 16. From the same group of patients, we excluded three patients with diffuse bone metastases and those who previously received external beam radiation therapy on the bone marrow regions. Thus, a total of 20 patients fulfilled the inclusion criteria for this analysis (Table S3). Figure 4A (upper) represents the baseline ^18^F-FDG-PET scan (left) and the follow-up ^18^F-FDG-PET scan (right) after the initiation of CIT, and the clearly enhanced ^18^F-FDG uptake within the bone was a consequence of successful treatment. Figure 4A (lower) represents the baseline ^18^F-FDG-PET scan (left) and the follow-up ^18^F-FDG-PET scan (right) after the initiation of CIT, indicating rather reduced ^18^F-FDG uptake within the bone is a consequence of CIT failure. Patients within the responding group revealed significantly higher baseline whole-body TBR values than the non-responding group (p<0.05; Figure 4B). Importantly, this difference was present in the axial bones (p<0.01) but not in the appendicular bones (Figure 4C). After CIT, the whole-body TBR values increased by 19.0±6.8 % in responders (p<0.01) but slightly decreased in non-responders (-5.9±4.8 %; Figure 4D). In the axial skeleton, the TBR increased by 16.7±5.0 % in the responders, whereas the uptake ratio decreased in the non-responders (-9.3±4.4%, p<0.01; Figure 4E). In the appendicular bones, we observed a trend towards CIT-treated responders having increased uptake, while the uptake of the non-responders remained relatively stable (Figure 4E).

After excluding patients with bone metastasis, we obtained comparable results for the responders (n=8) and non-responders (n=4), although the differences between responders and non-responders were not significant (Figure S2A/B). The percentage change reveals significant differences between responders and non-responders (n=12; Figure S2C/D).

### Retrospective analysis of blood parameters

After the onset of CIT-treatment, the routine tumor marker S100 and LDH increased in non-responders but decreased in responders (Figure S3A). Surprisingly, we observed a trend towards an elevation of the relative eosinophil count in non-responders but a decline in responders, whereas the relative lymphocyte counts declined in either responders or non-responders (Figure S3B).

## Discussion

As immune therapy is becoming widely applied in the clinics, the evaluation of therapy response is becoming tremendously important. Classically, the effect of cancer therapies is quantified by categories such as lesion size or ^18^F-FDG uptake, which are reflected by the Response Evaluation Criteria in Solid Tumors (RECIST; 18) guidelines. As immune responses can be accompanied by massive glucose consumption or an increase in the volumes of the tumors or lymphatic organs, the interpretation of clinical ^18^F-FDG-PET/CT data is hampered by CIT.

In contrast to conventional chemotherapy, cancer immune therapy can be correlated with survival benefits for patients even after an increase in total tumor burden or in the presence of new lesions 19. This led to the development of new response criteria, such as iRECIST (immune related RECIST) or PERCIMT (PET response criteria for the evaluation of immunotherapy), that consider factors like pseudo progression 20, 21. Dercle et al. analyzed the imaging patterns after three months of PD-1 mAb treatment in patients with refractory Hodgkin lymphoma and found that five of the 16 patients (31 %) exhibited new imaging patterns related to PD-1 mAb therapy that did not fit to the RECIST or Lymphoma Response to Immunomodulatory Therapy Criteria (LYRIC) criteria 22. Additionally, the authors observed an increase in splenic ^18^F-FDG uptake the in Hodgkin lymphoma patients receiving PD-1 mAb therapy 22. Regarding solid tumors, only reports about single cases of melanoma patients describe the ^18^F-FDG uptake in secondary lymphoid organs, such as the spleen or lymph nodes, after cancer immunotherapy 23, 24.

Here, our translational study of the metabolic patterns in response to CIT in solid tumors was able to identify an increase in splenic glucose metabolism in an experimental murine model of insular carcinoma, which was accompanied by decreased germinal centers of B and T cells but by increased numbers of MPO^+^ neutrophils, compared to those in response to the sham treatment. Nevertheless, our retrospective analysis of the clinical PET/CT data could not confirm that the splenic ^18^F-FDG uptake of patients with metastasized melanoma was significantly enhanced (See Supplementary Discussion 1).

In the RIP1-Tag2 mouse model the blood glucose level (BGL) declines with progressing insulinomas due to its insulin production. Nevertheless, the changes in insulin secretion are not explaining our results regarding the ^18^F-FDG uptake of the spleen, as we would expect a higher glucose uptake in the spleen of mice with larger tumors (sham treatment) if the spleen uptake would be insulin dependent.

As the spleen volume is reduced dramatically in sham-treated mice while the ^18^F-FDG uptake is only slightly decreased, the measured differences in the splenic ^18^F-FDG uptake between sham- and CIT-treated mice are not simply an effect of the changes in the spleen volumes.

In our experiments, flow cytometry revealed reduced populations of splenic CD3^+^ T cells as a consequence of combo- or CIT-treatment (Figure 1E, 2F) and a trend towards some mice having an increased presence of splenic neutrophils (Figure 1F). Immunohistochemistry depicted the expansion of the red pulp with a larger number of neutrophils as well as atrophy of the white pulp with fewer germinal centers after combo-treatment than after sham treatment (Figure 1G), which is in line with the flow cytometry data. Consequently, additional studies are needed to investigate the correlation of these findings to the effect of immunotherapy. Thus, an elevated infiltration of splenic neutrophils, which are highly active metabolically, could potentially explain the high splenic glucose metabolism observed by ^18^F-FDG-PET in our preclinical experiments. Both, activated neutrophils and T cells exhibit a strongly elevated glucose metabolism, but the neutrophils possess less mitochondria than other immune cells and are therefore highly dependent on anaerobic glycolysis 25, 26. Thus, the elevated ^18^F-FDG-uptake in the spleens of experimental mice with immunotherapy might be the consequence of the strongly elevated number of neutrophils and not T cells, which were decreasing in the spleen tissue of combo treated mice (Figure 1G). This opens new insights into the effects of an immunotherapeutic approach on the systemic immune response in a secondary lymphatic (the spleen) which was to date not reported elsewhere.

In our retrospective analysis of the clinical data, ^18^F-FDG-PET scans revealed only a slight increase in the glucose metabolism of the spleen in some patients, but there were no differences between responders and non-responders. Notably, patients in clinical practice are exposed to many different environmental stimuli that could potentially interact with the systemic immune response to CIT. Additionally, the unknown temporal dynamics of the immune response under CIT and the different time points of the ^18^F-FDG-PET scans in our retrospective study may have influenced the results.

Schwarzenberger et al. observed extramedullary splenic granulopoiesis and erythropoiesis after stimulation with IL-17 in mice 27. In contrast, granulopoiesis in humans switches from the liver, thymus, and spleen to the BM in the early stages of embryogenesis 38. This change may explain the different results we obtained from our preclinical experiments on mice and our retrospective analysis of clinical PET/CT data.

Thus, we focused on the BM as a primary lymphoid organ, especially since numerous studies have indicated that sufficient hematopoiesis is a prerequisite for successful cancer immunotherapy 28. The BM is the most important hematopoietic organ in adults for innate and adaptive immune cells and an important survival niche for naive and memory T cells, plasma cells, regulatory T cells and myeloid cells such as MDSCs 39. Increased hematopoiesis requires an enhanced metabolic rate in the BM, which leads to a high glucose uptake on the ^18^F-FDG PET scan 29-32. Furthermore, increased hematopoiesis induces an expansion of the active BM to the appendicular bones 21, 24. Unfortunately, the skeletons of mice were not able to be examined by ^18^F-FDG-PET in the same manner as the patient skeletons due to the limited spatial resolution of PET. Nevertheless, flow cytometry analysis of the BM revealed that CIT might induce the activation of T cells (Figure 1H, left) and that Tag2-Th1 cells might induce the activation of CD3-negative cells. (Figure 1H, right).

In our retrospective analysis of the clinical ^18^F-FDG-PET data, the glucose metabolism in the BM was significantly increased in patients with a clinical response to CIT compared to patients without a response to CIT (Figure 4B). In the axial skeleton, this effect was observed before the onset of treatment and in the follow-up examination, while in the appendicular skeleton, only a slight trend was noticeable in the follow-up ^18^F-FDG-PET scan; this difference could be due to an expansion of the active BM.

In the axial skeleton of responders, the ^18^F-FDG uptake in the BM increased significantly in the follow-up PET scan when compared to the baseline PET scan prior to onset of CIT. In sharp contrast, we observed a decreased ^18^F-FDG uptake in the BM of non-responders on the follow-up scan compared to the baseline scan (Figure 4E). A systematic review that included 12 studies with over 2,500 cancer patients who underwent ^18^F-FDG-PET examinations revealed a correlation between ^18^F-FDG uptake in the tumor and in the BM as well as the blood leukocyte count, which suggests a close relationship between the systemic inflammatory response involving BM activation and the tumor microenvironment 33. In our melanoma patient cohort, we observed a trend towards a high blood eosinophil count in non-responders as a consequence of CIT, but there were no significant differences in the relative neutrophil and lymphocyte counts between responders and non-responders (Figure S2). Whether our results of enhanced ^18^F-FDG uptake in the BM correlate with prognostic factors such as eosinophil count is unclear as the predictive power of our clinical study was limited due to the retrospective nature of the analysis and the relatively small number of patients. Weide et al. reported that elevated blood eosinophil counts prior to CIT might represent a positive prognostic factor in melanoma patients 28.

Novel and more sophisticated noninvasive *in vivo* molecular imaging methods using radiolabeled antibodies or immune cells are in preclinical development, and these methods inherently have a higher specificity than ^18^F-FDG-PET for the characterization of immune responses 34-37. Nevertheless, these approaches lack the possibility of immediate clinical translation due to regulatory, organizational, or economic issues, unlike ^18^F-FDG-PET, which is already part of the staging routine in many cancer types.

## Conclusions

Here, we present the first translational ^18^F-FDG-PET study on the impact of CIT in solid tumors on metabolic patterns in the BM and spleen. Thus, non-invasive *in vivo* ^18^F-FDG-PET/CT imaging of the glucose metabolism in the lymphatic organs might represent a powerful preclinical and clinical tool to discriminate responders from non-responders.

## Abbreviations

^18^F-FDG: 2-[^18^F]fluoro-2-deoxy-D-glucose
BGL: blood glucose level
BM: bone marrow
C3H: C3H mouse strain
CBC: complete blood count
CIT: checkpoint inhibitor therapy
CT: x-ray computed tomography
CTLA-4: cytotoxic T-lymphocyte-associated protein-4
GLUT1: transmembrane glucose transporter type 1
IFN-γ: interferon gamma
LAG-3: lymphocyte activation gene-3
LDH: lactate dehydrogenase
MRI: magnetic resonance imaging
mAbs: monoclonal antibodies
MACS: magnetic-activated cell sorting
MDSCs: myeloid derived suppressor cells
MPO: myeloperoxidase
PBS: phosphate-buffered saline
PD-1: programmed death-1
PD-L1: programmed death ligand 1
PET: positron emission tomography
RECIST: Response Evaluation Criteria in Solid Tumors
RIP1: rat insulin promotor
RIP1-Tag2: RIP1-Tag2 mice
SUV: standardized uptake value
Tag2: large T antigen
Tag2-TCR: Tag2-TCR mice
Tag2-Th1: IFN-γ-producing large T antigen (tumor antigen) -specific Th1 cells
TCR: T cell receptor
TBR: target-to-background ratio
VOI: volume of interest
