# Supplementary material for: Cancer immunotherapy is accompanied by distinct metabolic patterns in primary and secondary lymphoid organs observed by non-invasive in vivo 18F-FDG-PET
Source: Theranostics. 2020 Jan 1;10(2):925–37. doi: 10.7150/thno.35989 (PMC6929998; doi:10.7150/thno.35989)
Supplement: Supplementary file 1 — Supplementary figures and tables. [file thnov10p0925s1.pdf]

Cancer immunotherapy is accompanied by distinct metabolic patterns in primary and secondary lymphoid organs observed by non-invasive *in vivo* ^{18}F-FDG-PET

Johannes Schwenck[1,2,3*], Barbara Schörg[2*], Francesco Fiz[1,4], Dominik Sonanini[2,5], Andrea Forschner[6], Thomas Eigentler[6], Benjamin Weide[6], Manuela Martella[7], Irene Gonzalez-Menendez[3,7], Cristina Campi[8], Gianmario Sambuceti[9], Ferdinand Seith[10], Leticia Quintanilla-Martinez[3,7], Claus Garbe[6], Christina Pfannenberg[10], Martin Röcken[3,6,11], Christian la Fougere[1,3,11], Bernd J Pichler[2,3,11], Manfred Kneilling[2,3,6$]

[1] Department of Nuclear Medicine and Clinical Molecular Imaging, Eberhard Karls University, 72076 Tübingen, Germany
[2] Werner Siemens Imaging Center, Department of Preclinical Imaging and Radiopharmacy, Eberhard Karls University, 72076 Tübingen, Germany
[3] Cluster of Excellence iFIT (EXC 2180) "Image-Guided and Functionally Instructed Tumor Therapies", Eberhard Karls University, 72076 Tübingen, Germany
[4] Department of Internal Medicine, University of Genoa, Italy
[5] Department of Internal Medicine II, Eberhard Karls University, 72076 Tübingen, Germany
[6] Department of Dermatology, Eberhard Karls University, 72076 Tübingen, Germany
[7] Institute of Pathology and Neuropathology and Comprehensive Cancer Center Tübingen, Eberhard Karls University, 72076 Tübingen, Germany
[8] Department of Mathematics "Tullio Levi-Civita", University of Padua, Italy
[9] Nuclear Medicine Unit, Department of Health Sciences, University of Genoa, Italy
[10] Department of Diagnostic and Interventional Radiology, Eberhard Karls University, 72076 Tübingen, Germany
[11] German Cancer Consortium (DKTK), German Cancer Research Center (DKFZ) Partner Site Tübingen, 72076 Tübingen, Germany

* Contributed equally

[$] Corresponding author email: manfred.kneilling@med.uni-tuebingen.de
telephone: +49-7071-29-83427
fax: +49-7071-29-4451

Short title: Metabolic patterns in immunotherapy responders

Supplementary Materials and Methods

Generation of tumor antigen-specific Th1 (Tag2-Th1) cells

Tumor antigen-specific Th1 cells were generated as previously described [1]. Briefly, $CD4^+$ T cells were isolated from the spleens and lymph nodes of transgenic Tag2-TCR mice and separated via magnetic-activated cell sorting (MACS) according to the manufacturer's protocol (Milteny Biotec GmbH, Bergisch Gladbach, Germany). The cells were co-cultivated together with irradiated (30 Gy) antigen presenting cells, CPG oligonucleotide (ODN1668; Eurofins Genomics Germany GmbH, Ebersberg, Germany), anti-IL-4 mAbs (EMC Microcollections GmbH).

Immunotherapy in RIP1-Tag2 mice

The first cohort of ten- to eleven-week-old RIP1-Tag2 mice (n=5 per treatment group) with advanced insular cell carcinomas was treated according to the treatment scheme in Fig. 1A. Briefly, combo-treated mice received weekly injections (i.p.) of $1x10^6$ Tag2-Th1 cells followed by anti-PD-L1 plus anti-LAG-3 mAbs (first injection: 500 µg/mAb, following: 200 µg/mAb; BioXcell, NH, USA) 24 h after whole-body radiation (2 Gy). Control mice received either isotype-matched IgG (Iso) mAbs (BioXcell, NH, USA) and/or PBS instead of T cells. (CIT: mAbs+PBS, Th1: Iso+Tag2-Th1, sham: Iso+PBS).

A second cohort of 10- to 11-week-old RIP1-Tag2 mice mimicked the clinical conditions and exclusively received anti-PD-L1 and anti-LAG-3 mAbs (i.p.), importantly without the initial 2 Gy whole-body radiation (CIT, n=8) (Fig. 2A). Sham-treated mice received Iso mAb-injections twice weekly (i.p.; n=8).

Supplementary Discussion

Although a nonessential organ, the spleen has important functions as a reservoir and as a site of priming and proliferation for lymphocytes [2]. Additionally, the spleen is involved in the induction of immune tolerance as well as in the removal of aged or dysfunctional blood cells [2, 3]. Undoubtedly, the spleen is involved in the response to systemic inflammatory stimuli, such as lipopolysaccharides or RNA vaccinations, which is observable by the enhanced splenic ^{18}F-FDG-PET uptake [4, 5]. In the endogenous insular cell carcinoma model, we observed an elevated ^{18}F-FDG tracer uptake in the spleen in response to PD-L1 mAbs and LAG-3 mAbs with or without Tag2-Th1 cells, which reflects the enhanced glucose metabolism (Fig. 1C, D and 2C, D). In addition, we observed a trend towards some patients with metastatic melanoma having increased splenic glucose metabolism in response to CIT, but no significant differences were found between responders and non-responders (Fig. 3D). The spleen is important for the immune tolerance of tumors as splenic myeloid-derived suppressor cells (MDSCs) play a major role in suppressing the antitumoral immune responses [3, 6]. However, little is known about how the spleen is involved in overcoming immune tolerance by cancer immune therapy. Zhuang et al. revealed an increase in the number of splenic effector memory T cells by applying an effective combination therapy that consisted of local radiotherapy, intra-tumoral cytosine-phosphorothioate-guanine, and systemic PD-1 mAb administration in an experimental lung cancer model [7]. The activation of T cells requires sufficient glucose uptake via GLUT1, which can be measured by ^{18}F-FDG-PET uptake [8-12]. Using ^{18}F-labeled 2'-deoxycytidine, Radu et al. revealed significant uptake in the spleen and lymph nodes as a consequence of an anti-tumoral immune response [13].

Supplementary References

1. Braumuller H, Wieder T, Brenner E, Assmann S, Hahn M, Alkhaled M, et al. T-helper-1-cell cytokines drive cancer into senescence. Nature. 2013; 494: 361-5.
2. Bronte V, Pittet MJ. The spleen in local and systemic regulation of immunity. Immunity. 2013; 39: 806-18.
3. Ugel S, Peranzoni E, Desantis G, Chioda M, Walter S, Weinschenk T, et al. Immune tolerance to tumor antigens occurs in a specialized environment of the spleen. Cell Rep. 2012; 2: 628-39.
4. Pektor S, Hilscher L, Walzer KC, Miederer I, Bausbacher N, Loquai C, et al. In vivo imaging of the immune response upon systemic RNA cancer vaccination by FDG-PET. EJNMMI Res. 2018; 8: 80.
5. Pektor S, Bausbacher N, Otto G, Lawaczeck L, Grabbe S, Schreckenberger M, et al. Toll like receptor mediated immune stimulation can be visualized in vivo by [(18)F]FDG-PET. Nucl Med Biol. 2016; 43: 651-60.
6. Li B, Zhang S, Huang N, Chen H, Wang P, Li J, et al. Dynamics of the spleen and its significance in a murine H22 orthotopic hepatoma model. Exp Biol Med (Maywood). 2016; 241: 863-72.
7. Zhuang Y, Li S, Wang H, Pi J, Xing Y, Li G. PD-1 blockade enhances radio-immunotherapy efficacy in murine tumor models. J Cancer Res Clin Oncol. 2018; 144: 1909-20.
8. Wofford JA, Wieman HL, Jacobs SR, Zhao Y, Rathmell JC. IL-7 promotes Glut1 trafficking and glucose uptake via STAT5-mediated activation of Akt to support T-cell survival. Blood. 2008; 111: 2101-11.
9. Cammann C, Rath A, Reichl U, Lingel H, Brunner-Weinzierl M, Simeoni L, et al. Early changes in the metabolic profile of activated CD8(+) T cells. BMC Cell Biol. 2016; 17: 28.
10. Saucillo DC, Gerriets VA, Sheng J, Rathmell JC, Maciver NJ. Leptin metabolically licenses T cells for activation to link nutrition and immunity. J Immunol. 2014; 192: 136-44.
11. Jacobs SR, Herman CE, Maciver NJ, Wofford JA, Wieman HL, Hammen JJ, et al. Glucose uptake is limiting in T cell activation and requires CD28-mediated Akt-dependent and independent pathways. J Immunol. 2008; 180: 4476-86.
12. Cretenet G, Clerc I, Matias M, Loisel S, Craveiro M, Oburoglu L, et al. Cell surface Glut1 levels distinguish human CD4 and CD8 T lymphocyte subsets with distinct effector functions. Sci Rep. 2016; 6: 24129.
13. Radu CG, Shu CJ, Nair-Gill E, Shelly SM, Barrio JR, Satyamurthy N, et al. Molecular imaging of lymphoid organs and immune activation by positron emission tomography with a new [18F]-labeled 2'-deoxycytidine analog. Nat Med. 2008; 14: 783-8.

Supplementary Figures and Tables

Figure S1: Patient selection

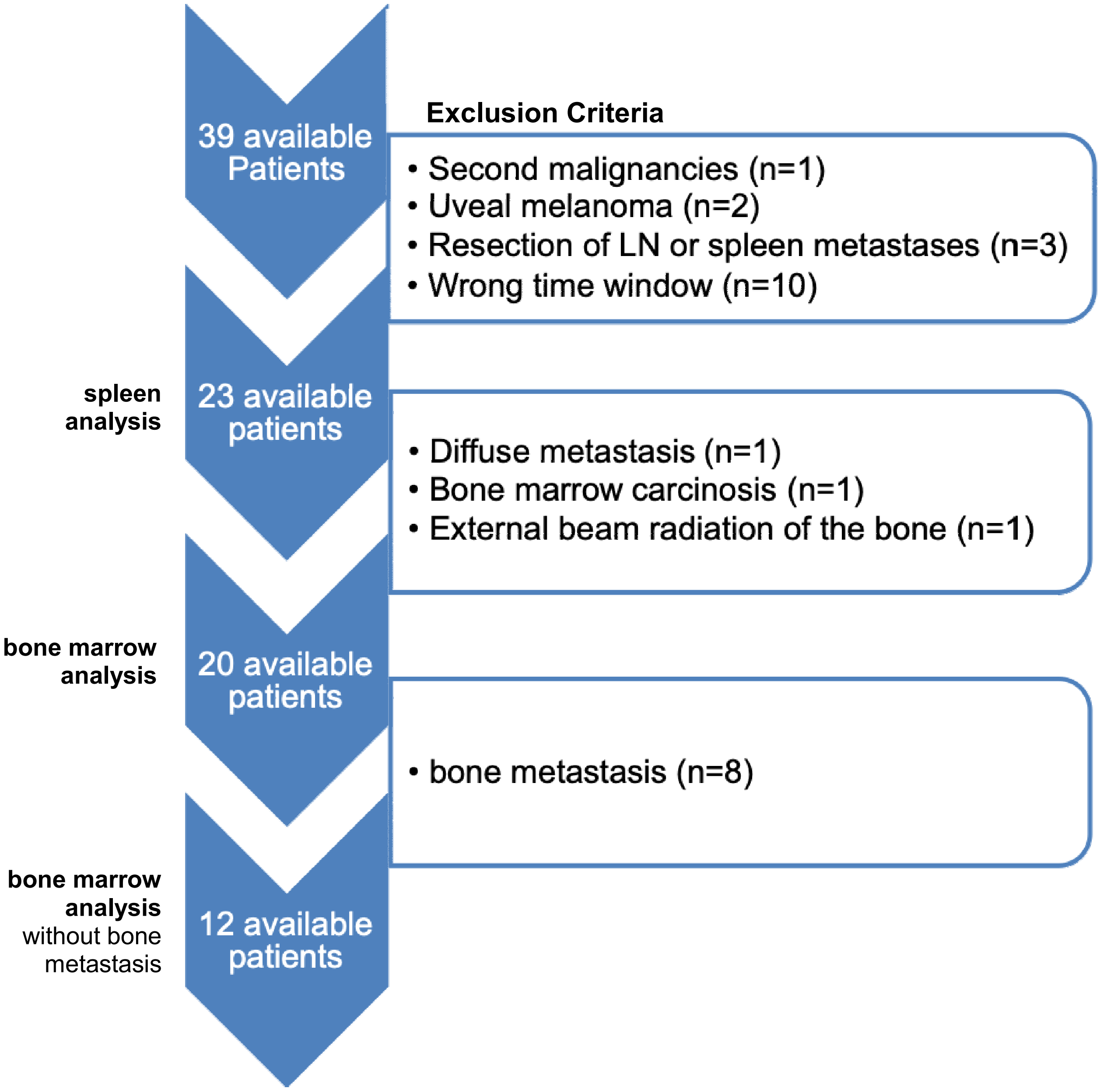

In total, the data from 39 patients with metastatic melanoma and their ^{18}F-FDG-PET/CT were available. For the analysis of the spleen, patients who had a second malignant disease, surgical resections of metastasis, splenic metastasis and uveal melanomas were excluded. In total, 23 patients with ^{18}F-FDG-PET scans within 50 days before and 125 days after the start of therapy were identified for further analysis. For the analysis of the bone marrow, we excluded patients with diffuse

bone metastases, bone marrow carcinosis and previous external beam radiation therapy on bone marrow regions from the analysis of the bone marrow.

Figure S2: Retrospective analysis of the bone marrow excluding the patients with bone metastases

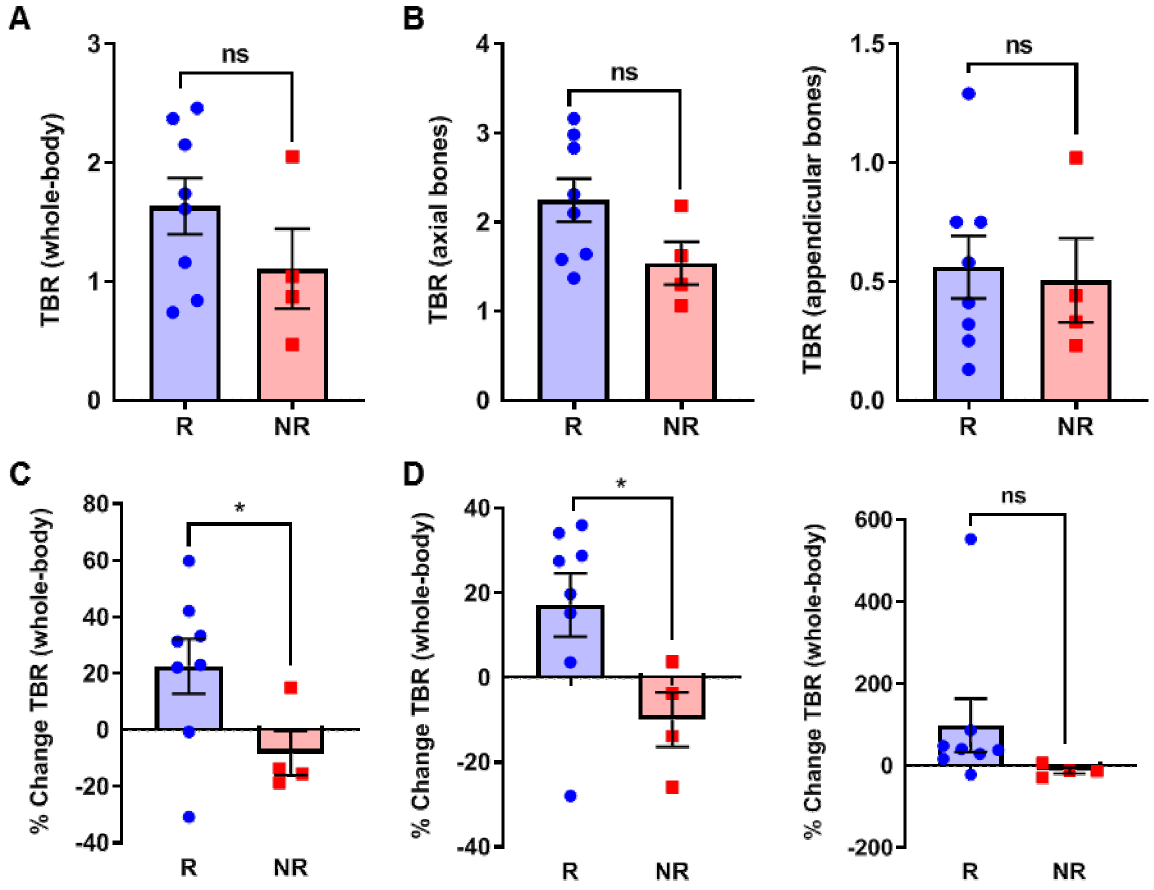

(A) TBRs after excluding the five responders and three non-responders with bone metastases at the baseline scan. (B) TBRs in the axial and appendicular bones of patients who responded or did not respond to CIT. (C) % change between the baseline and follow-up PET/CT scans in the whole-body TBR. (D) % change of the TBR after the separate analysis of the axial and appendicular bones. Data are expressed as the mean ± SEM (responder n=8; non-responder n=4). Each data point represents one patient (*P<0.05, ns = not significant).

Figure S3: Retrospective analysis of the blood parameters

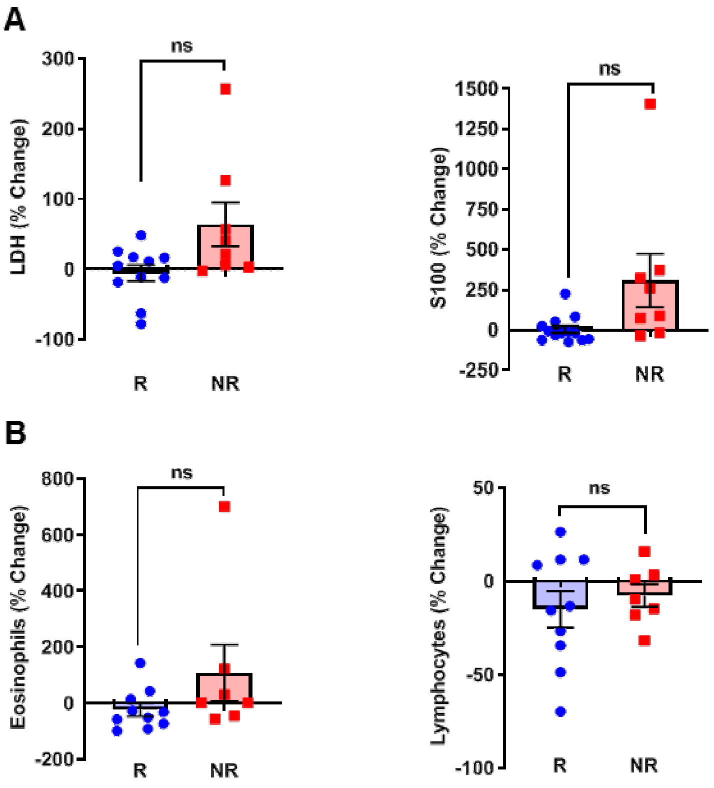

(A) The % change of the malignant melanoma tumor markers LDH (5.8±11.3 % vs. 63.2±31.4) and S100 (2.9±22.6 % vs. 307.9±165.7 %) in the blood of responders and non-responders between the baseline and follow-up PET/CT scans. (B) Relative eosinophil counts (24.4±23.2 % vs. 106.9±101.3 %) and relative lymphocyte counts in responders and non-responders (-15.2±9.6 % vs. -7.8±6.0 %); Data are expressed as the mean ± SEM (LDH responders n=11 non-responders n=8; S100 responders n=13 non-responders n=8; eosinophils/lymphocytes responders n=10 non-responders n=7). Each data point represents one patient (ns = not significant).

Table S1: Clinical ^{18}F-FDG-PET/CT data from patients with metastatic melanoma

**Responder**	**Age**	**Sex**	**Checkpoint inhibitor**	**Days between baseline<->therapy start**	**Days between therapy start<->followup**	
**1**	50	m	Nivolumab	28	96	
**2**	77	m	Nivolumab	19	121	
**3**	52	m	Ipilimumab	1	77	
**4**	62	m	Ipilimumab	6	43	
**5**	74	m	Pembrolizumab	5	86	
**6**	75	f	Pembrolizumab	13	96	
**7**	94	f	Pembrolizumab	26	88	
**8**	66	m	Ipilimumab	13	66	
**9**	84	m	Pembrolizumab	20	83	
**10**	62	f	Ipilimumab	50	89	
**11**	60	m	Pembrolizumab	7	84	
**12**	75	m	Pembrolizumab	20	90	
**13**	85	f	Nivolumab	5	85	
**14**	53	f	Ipilimumab+ Pembrolizumab	4	82	
**Non-responder**						
**15**	59	f	Ipilimumab	14	55	
**16**	77	m	Ipilimumab	23	104	
**17**	75	f	Ipilimumab	35	74	
**18**	80	f	Pembrolizumab	35	88	
**19**	75	m	Pembrolizumab	5	87	
**20**	35	m	Nivolumab	8	81	
**21**	72	f	Ipilimumab	14	58	
**22**	76	m	Pembrolizumab	27	91	
**23**	59	f	Ipilimumab	21	82	
**Excluded**						**Reasons for exclusion**
**24**	73	m	Ipilimumab	20	**131**	Follow up too late
**25**	58	f	Pembrolizumab	13	76	Primary lung cancer
**26**	68	f	Ipilimumab	**104**	108	Baseline too early
**27**	66	m	Nivolumab	**89**	76	Baseline too early
**28**	21	f	Ipilimumab	13	70	Resection of bone metastasis, radiation
**29**	59	f	Ipilimumab	24	**317**	Follow up too late
**30**	39	m	Ipilimumab	35	**191**	Follow up too late

**31**	51	m	Nivolumab	**112**	**376**	Follow up too late
**32**	56	m	Pembrolizumab	16	91	Lymph node resection
**33**	77	m	Pembrolizumab	17	**168**	Follow up too late
**34**	71	m	Nivolumab	34	**267**	Follow up too late
**35**	43	m	Pembrolizumab	**74**	93	Baseline too early
**36**	72	f	Pembrolizumab	30	91	Uveal melanoma, Bone metastasis, radiatio
**37**	74	f	Pembrolizumab	23	98	Splenic metastasis
**38**	67	m	Pembrolizumab	1	86	Uveal melanoma, B-cell lymphoma
**39**	61	m	Pembrolizumab	**76**	103	Baseline too early

m, male; f, female

Table S2: Clinical splenic ^{18}F-FDG-PET/CT data

Responder	Checkpoint inhibitor	Days between baseline<->therapy start	Days between therapy start<->follow up	baseline SUVmean	follow up SUVmean	% change SUVmean
1	Nivolumab	28	96	2,01	1,95	-2,99
2	Nivolumab	19	121	1,71	1,97	15,20
3	Ipilimumab	1	77	1,51	1,41	-6,62
4	Ipilimumab	6	43	1,86	1,86	0,00
5	Pembrolizumab	5	86	2,11	1,05	-50,24
6	Pembrolizumab	13	96	2,16	1,69	-21,76
7	Pembrolizumab	26	88	1,59	1,41	-11,32
8	Ipilimumab	13	66	1,57	1,74	10,83
9	Pembrolizumab	20	83	1,73	1,5	-13,29
10	Ipilimumab	50	89	1,48	1,86	25,68
11	Pembrolizumab	7	84	1,04	2,16	107,69
12	Pembrolizumab	20	90	2,05	1,75	-14,63
13	Nivolumab	5	85	1,89	1,95	3,17
14	Ipilimumab+ Pembrolizumab	4	82	1,62	2,86	32,00
Non-responder						
15	Ipilimumab	14	55	1,96	1,63	-16,84
16	Ipilimumab	23	104	1,51	1,51	0,00
17	Ipilimumab	35	74	1,55	1,63	5,16
18	Pembrolizumab	35	88	1,67	2,08	24,55
19	Pembrolizumab	5	87	1,46	1,38	-5,48
20	Nivolumab	8	81	1,57	1,73	10,19
21	Ipilimumab	14	58	1,48	1,65	11,49
22	Pembrolizumab	27	91	1,86	1,47	-20,97
23	Ipilimumab	21	82	2,21	2,16	-2,26

SUV, standard uptake value

Table S3: Clinical bone marrow ^{18}F-FDG-PET/CT data

Responder	TBR Baseline whole Body	TBR Baseline axial	TBR Baseline appen-dicular	TBR Follow up whole Body	TBR Follow up axial	TBR Follow up appen-dicular	% change wholebody	% change axial	% change appen-dicular	
1	2,37	3,16	1,29	1,64	2,27	1,00	-30,91	-28,03	-22,45	
2	1,20	1,50	0,40	1,10	1,50	0,30	-8,33	0,00	-25,00	Bone metastasis
3	2,46	2,98	0,75	3,00	3,44	1,38	22,07	15,20	85,29	
4	2,83	3,33	2,17	3,83	4,47	2,98	35,17	34,04	37,48	Bone metastasis
5	1,52	1,92	1,21	1,50	2,25	0,63	-1,00	17,24	-48,44	Bone metastasis
6	1,16	1,58	0,13	1,85	2,15	0,85	59,72	35,94	551,54	
7	0,74	1,37	0,32	1,05	1,74	0,47	42,03	27,46	47,29	
8	1,80	2,46	0,82	1,94	2,54	0,75	7,60	3,18	-8,96	Bone metastasis
9	2,15	2,83	0,58	2,13	2,93	0,80	-0,78	3,53	37,14	
10	0,84	2,31	0,25	1,12	2,77	0,29	33,25	19,70	15,94	
11	1,61	1,64	0,41	2,12	2,21	0,58	31,30	34,10	40,33	
12	1,60	2,00	0,67	2,14	2,53	1,14	33,93	26,43	71,43	Bone metastasis
13										bone marrow carcinosis
14	1,74	2,10	0,75	2,14	2,71	0,96	22,94	28,75	27,73	
Non-responder										
15										Diffuse bone metastases
16										Radiation
17	0,87	1,06	0,44	0,70	1,02	0,39	-18,72	-3,77	-12,11	
18	0,47	1,62	0,33	0,54	1,40	0,35	14,94	-13,87	6,25	
19	2,05	2,18	1,02	1,76	1,62	0,88	-13,73	-25,86	-13,73	
20	1,00	1,36	0,41	0,88	1,23	0,34	-12,41	-9,98	-15,71	Bone metastasis
21	1,04	1,30	0,23	0,88	1,35	0,16	-15,53	3,76	-29,09	
22	1,02	1,31	0,27	1,00	1,37	0,67	-1,90	4,70	146,54	Bone metastasis
23	1,46	1,88	1,54	1,54	1,50	1,07	6,00	-20,23	-30,38	Bone metastasis

TBR, target-to-background ratio
